# Accurate Single-Particle Tracking and Diffusion Measurement in Freestanding Lipid Bilayers and Model Membranes

**DOI:** 10.1021/acs.analchem.5c05037

**Published:** 2025-12-08

**Authors:** Lily Anne Van Ye, Richard D. Michael, Joshua J. Meyer, Sophia M. Peña, Derek J. Bailey, Lisa M. Keranen-Burden, Daniel L. Burden

**Affiliations:** 8560 Wheaton College, Dept. of Chemistry, Wheaton, Illinois 60187, United States

## Abstract

Single-particle tracking (SPT) in lipid bilayers offers a powerful means to investigate the dynamic interaction of individual molecules in a variety of model membranes. However, accurate diffusion characterization in freestanding bilayers remains challenging due to membrane fragility, the potential for curvature, limited optical signal-to-background ratio, and the practical need for label concentrations that do not approximate infinite dilution. Here, we report a dual-mode fluorescence microscope configuration for bilayers formed on a microelectrode cavity array (MECAopto-inv chip) that combines widefield SPT with confocal fluorescence correlation spectroscopy (FCS). By using FCS to calibrate the SPT algorithm, we demonstrate improved measurement accuracy for diffusion constants. Importantly, we show that proper calibration resists measurement bias at high fluorescent particle concentrations. Supporting simulations confirm the robustness of this approach. When calibrated with FCS and corrected for blur, our measurements help resolve the variability in diffusion constants reported in prior literature. Furthermore, the strategy is broadly applicable to any planar bilayer system. The method provides a rigorous, quantitative benchmark for future studies involving nanopores, membrane protein assemblies, and ion-channel transport in lipid bilayers, especially when both optical and electrical information is desired.

## Graphical abstract

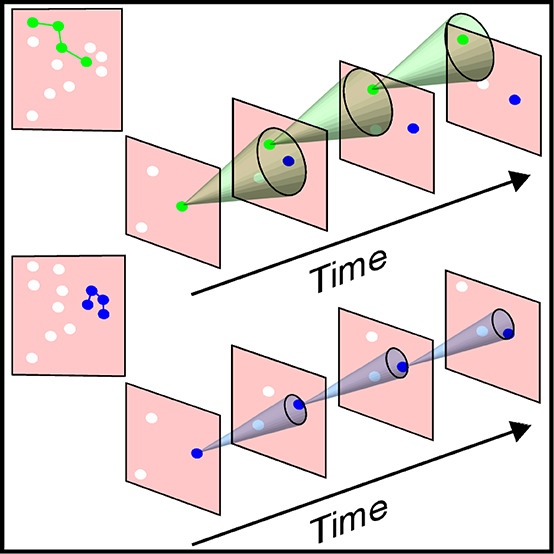

## Introduction

Optical detection of individual fluorescent molecules within biological membranes provides unique insight into the behavior of lipids, membrane proteins, glycolipids, and related components. Tracking molecular position over time provides the opportunity to observe movement, interactions, and changes in conformation. Many studies performed in supported lipid bilayers and hybrid lipid bilayers have been widely reported in the literature,^1–9^ where established methodologies allow for stable single-molecule measurements. In contrast, single-molecule optical studies within unsupported or freestanding planar bilayer systems are less common but growing in importance, as these systems offer a chemically simple environment that is free from substrate interactions.^9–15^ They are often formed from a two-component mixture of lipid and hydrocarbon solvent that is spread over an aperture immersed in water.^16,17^ This approach brings aqueous solution into direct contact with each side of the bilayer. Freestanding bilayers can also be formed with a high transmembrane electrical resistance, making them a platform of choice for studying the conductance of single ion channels and biological nanopores.

However, these structures present a number of optical measurement challenges, especially for single-chromophore detection and tracking.^1,17^ Historically, freestanding bilayers have been formed one bilayer at a time over individual apertures. Due to bilayer fragility and the complexity of sample positioning, experiments were often met with limited success. To address this difficulty, we employed a recently developed commercial device to interrogate multiple bilayers formed on a microelectrode cavity array chip.^18^ The parallel arrangement of freestanding bilayers within the chip mitigates the statistical failure rate associated with bilayer rupture and allows selective optical interrogation. The closed microcavity design also conveys added membrane stability in comparison to open configurations that expose the bilayer to atmospheric pressure gradients. Importantly, the chip facilitates simultaneous optical and transmembrane electrical recordings at the single-molecule level. In a recent study, we utilized the electrical features of microelectrode cavity arrays, in combination with a confocal microscope, to characterize the translocation of single dye molecules *through* individual nanopores.^19^ The study demonstrated the first direct measurement of electrically silent ion channel translocation events.

To our knowledge, the positional tracking of single molecules *within* freestanding bilayers formed on these chips has not yet been reported. The size and geometry of the microcavity, background autofluorescence from the chip, and the location of the microelectrodes places constraints on the single-molecule optical system that can be employed. In turn, the specific optical configuration determines parameters such as the laser illumination intensity, the camera exposure time, and the frame rate necessary for generating signal levels capable of tracking individual chromophores.

Additional measurement complexities arise from the unpredictable shape of freestanding bilayers suspended over closed microcavities. Commonly used formation techniques can result in bilayers that are slightly curved in either a concave or convex direction. Because single-particle tracking (SPT) algorithms assume planarity, curvature can introduce systematic bias in the determination of diffusion constants. Errors also arise if particle trajectories within the bilayer are inadequately defined and are exacerbated when the diffusion constant is large and the bilayer is crowded with trackable molecules. Accuracy under noninfinitely dilute conditions is particularly important for studies of multicomponent membrane proteins, ion channel assembly dynamics, pore-forming peptides, and ionophores, which are all possible using microelectrode cavity arrays. To begin countering these challenges, a method for ensuring the accuracy of SPT measurements in freestanding bilayers under a range of common experimental conditions is needed.

Here, we describe a dual confocal and widefield microscope configuration that standardizes SPT and diffusion characterization in freestanding bilayers formed on microelectrode cavity arrays. We use Fluorescence Correlation Spectroscopy (FCS) to optimize the maximum linking radius (MLR), which is a common input parameter for many SPT algorithms, and show that calibration provides improved accuracy. In addition, we explore the general issue of measurement errors over a range of fluorescently labeled lipid concentrations and diffusion constants. To test the validity of the dual-mode approach, we compare results to theoretical determinations of the MLR discussed by other investigators and to output from diffusion simulations that closely mimic the experimental apparatus. The findings lay groundwork for more accurate characterization of membrane proteins and nanopores in freestanding membranes. The same calibration protocol can also be applied for use with a variety of other model bilayers, including supported and hybrid bilayer systems.

## Materials and Methods

### Instrument Setup

All measurements were performed using an Orbit Mini bilayer workstation (Nanion Technologies, Munich, Germany) with a MECAopto-inv chip. The chip (Figure 1A) possesses 4 simultaneously accessible microwells, each with a ring-shaped Ag/AgCl electrode deposited on a thin cover glass (#1.5, 170 μm thick). Microwells are formed in a 25-μm thick SU8 polymer layer on top of the cover glass. Each 150-μm diameter microwell holds ∼ 440 pL of aqueous electrolyte solution. Microwells are located in the bottom of a single larger well (∼0.4 mL) that contains a Ag/AgCl electrode held at virtual ground.

**Figure 1:**
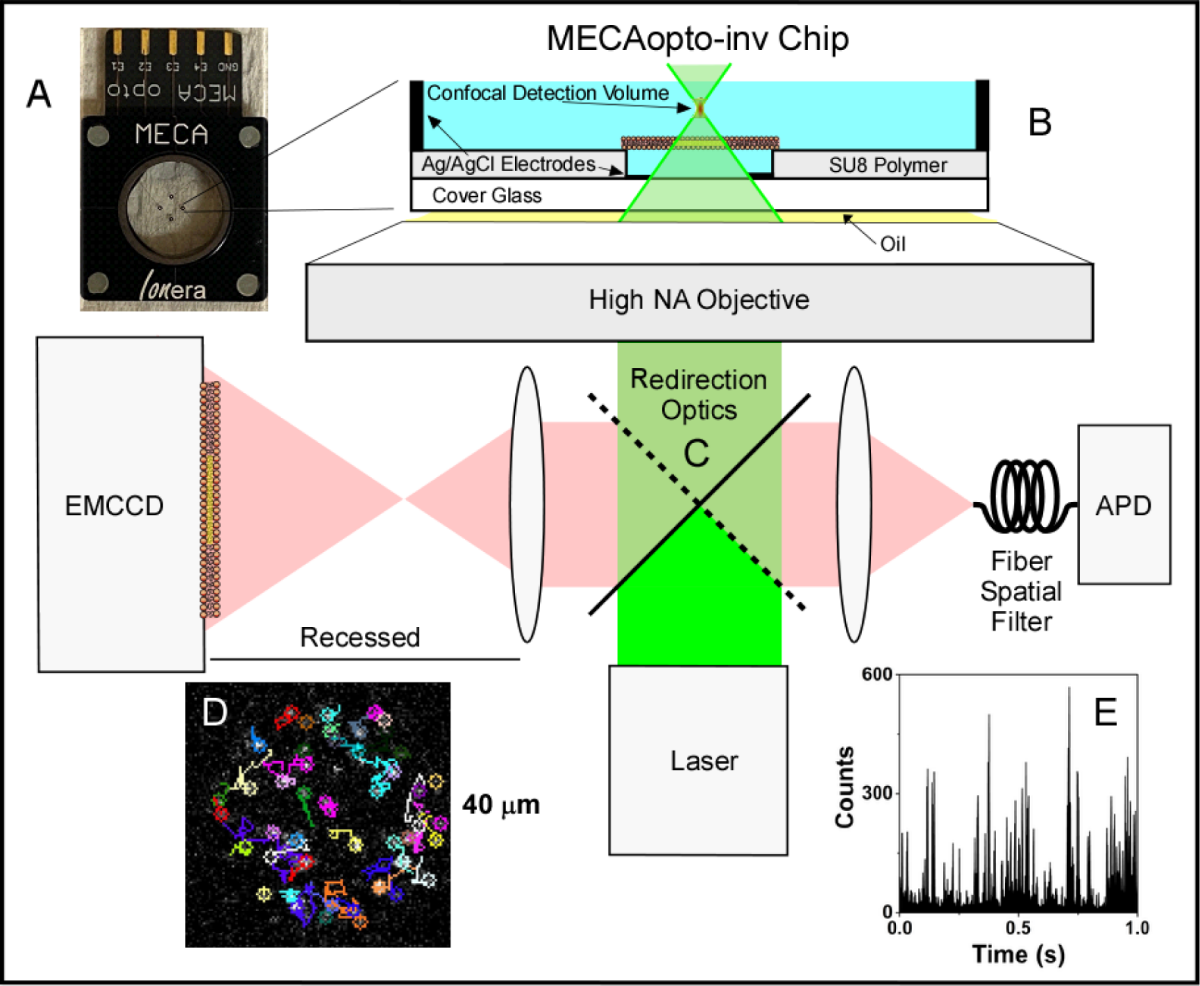
Dual confocal and widefield imaging for microelectrode cavity arrays. (A) Photo of the MECAopto-inv chip and (B) its position within the microscope optical system. (C) Switchable internal microscope optics enable the bilayer to be probed in either the confocal or widefield mode. (D) A single widefield frame of fluorescent spots from single molecules overlaid with local particle trajectories from the bilayer (≈0.035 particles μm^–2^). See Supplemental Video (S1). (E) Photon bursts acquired from the APD when the confocal detection volume is positioned on the bilayer.

The Orbit Mini and the MECAopto-inv chip were mounted on a Nikon Ti-E inverted microscope (Nikon, Tokyo, Japan). A motorized stage on the Ti-E allowed rapid repositioning between the four microwells. Confocal operation of the microscope employed a collimated 645 nm laser that entered the microscope through the rear port. A dichroic mirror (655 nm) directed the beam to the objective (Nikon 100x, Plan Apo TIRF, N.A. = 1.49), which focused the laser to a near diffraction-limited spot (measured diameter of 624 nm). The converging beam propagated through the cover glass just inside the opaque Ag/AgCl electrode ring and the SU8 layer defining the microwell (Figure 1B and C). The confocal detection volume was located ∼ 10 μm above the camera object plane. A bandpass emission filter (660 to 740 nm) was used to isolate fluorescence photons. A fiber optic spatial filter (100 μm diam., multimode), located in an output port of the microscope, was positioned in the conjugate focal plane and delivered fluorescence photons to an avalanche photodiode (APD) module (SPCM-AQHR, Excelitas, Pittsburgh, PA). Photocounts from the APD were registered continuously using a multichannel scalar (MCS, Ortek/Ametek, Oak Ridge, TN, Figure 1E). Autocorrelation curves were produced by a hardware autocorrelator (5000/600MultipleTau Digital Correlator, ALV, Langen, Germany).

Widefield imaging and single-particle tracking were facilitated by an electron multiplying charge-coupled device (EMCCD) camera (iXon, Andor/Oxford Instruments, Santa Barbara, CA) mounted in a second side port of the microscope. The camera mount was recessed from the body of the microscope to create a difference of ∼ 10 μm between the location of confocal detection volume and the object plane imaged by the camera. The converging laser created a circular illumination profile of the bilayer (∼37 μm diameter) that permitted detection and tracking of single molecules over an area of 1.1 × 10^3^ μm^2^ (Figure 1D). Switchable internal microscope optics (Figure 1C) allowed redirection of the light path. A piezo-driven axial controller on the objective turret allowed repositioning of the beam in the axial direction so that the bilayer could be probed using either confocal or widefield illumination.

### Membrane Formation, Characterization, and Electrical Monitoring

Planar lipid bilayer membranes were formed using the traditional painting technique.^16^ The membrane capacitance (typically 50 to 90 pF) was monitored over time to determine when the membranes were sufficiently thinned, indicating retreat of the solvent annulus to the outer perimeter (i.e., ∼ 15–25 μm) of the 150-μm diameter membrane. Typically, capacitances stabilized in a few minutes and yielded planar structures for optical measurement. Bilayers often remained intact for multiple hours, or until the experiment was terminated. In the event of membrane rupture or destabilization, the microscope could be quickly repositioned over an alternate microwell with an intact bilayer. All data were acquired on bilayers formed from a mixture of cyanine-5 labeled 1,2-distearoyl-*sn*-glycero-3-phosphoethanolamine (Cy5-DSPE, BroadPharm, San Diego, CA) and 1,2-diphytanoyl-*sn*-glycero-3-phosphocholine (DPhPC, Avanti Polar Lipids, Birmingham, AL) at mole ratios from 1:(0.5 to 10)­x10^6^ in octane (Millipore-Sigma, Milwaukee, WI) at 50 mg mL^–1^. Capacitance recordings of membranes formed over the microwell were acquired with Elements Data Reader 3 software (Elements, Cesena, Italy).

### Single Chromophore Signal Acquisition

The excitation laser power was optimized to generate signal-to-noise ratios capable of single-molecule detection while minimizing the effects of photobleaching and background. Excitation intensities differed depending on the mode of detection. Generally, we explored a range from 40 to 250 W cm^–2^ and 3 to 40 kW cm^–2^ in the widefield and confocal modes, respectively. All analyses shown here were performed on data collected at ∼ 120 W cm^–2^ for SPT and 5 kW cm^–2^ for FCS. Autocorrelation curves acquired at 5 kW cm^–2^ show negligible photobleaching. However, due to the extended exposure time in the widefield mode, photobleaching shortens the length of the chromophore trajectories in SPT data sets, even at the lowest illumination intensity. To facilitate FCS diffusion calibration of SPT data sets, both confocal and widefield data were acquired from the same bilayer. The EMCCD camera (512 × 512 pixels) collected fluorescence photons using a 40 ms exposure time (Δ*T_exp_*) under continuous illumination with 2 × 2 pixel binning, 10 MHz chip readout speed, and a gain multiplier of 900. This produced a corresponding frame rate of 24 frames s^–1^ (i.e., 42 ms interval, Δ*T*) and a spatial resolution of 336 nm pixel^–1^ after binning.

### Confocal Detection Volume Size Characterization

To determine the diffusion constant of the lipids from the FCS data, the confocal detection volume dimensions were carefully characterized. The full procedure for determining the dimensions have been reported earlier.^19^ The detection volume and radial dimension (*ω*_1_) were found to be 3.9 × 10^–15^ L and 312 nm, respectively.

### FCS Diffusion Analysis

The dual FCS/SPT configuration allowed autocorrelation of fluorescent lipid burst signatures from single molecules (Figure 1E). Autocorrelation plots were generated by placing the waist of the detection volume in the plane of the bilayer. Data were fit to eq 1, where *N* is the average number of dye molecules occupying the 2D detection region on the membrane, τ is time lag, and *τ_D_* is the average crossing time of a particle:

1 $$G_{2D}\left(\tau \right)=\left[\frac{1}{\left\langle N\right\rangle }\right]\cdot \left[{\left(1+\frac{\tau }{\tau_{D}}\right)}^{-1}\right]$$

In conjunction with the measured radius (*ω*_1_) of the confocal detection volume, we used *τ_D_* to calculate the diffusion constant (*D*):

2 $$D=\omega_{1}^{2}/\left(4\cdot \tau_{D}\right)$$

### Single Particle Tracking and Trajectory Analysis

Numerous software tools are available for particle detection, trajectory linking, and analysis.^20–26^ This study compared results from Particle Tracker (PT)^27^ and TrackMate (TM).^28^ Both are commonly employed in single-molecule fluorescence studies. Furthermore, these tools are available as conveniently accessible plugins to Fiji (ImageJ) and employ similar cost-matrix minimization algorithms for feature-based particle detection and tracking.

The squared displacement (*SD*) and mean squared displacement (*MSD*) for each time lag (Δ*t*) are computed as

3 $$\mathrm{MSD}\left(\Delta t\right)=\langle SD\left(\Delta t)\right\rangle =\left\langle r^{2}\left(\Delta t\right)\right\rangle =\left\langle {\left(x\left(t+\Delta t\right)-x\left(t\right)\right)}^{2}+{\left(y\left(t+\Delta t\right)-y\left(t\right)\right)}^{2}\right\rangle$$

This is related to the diffusion constant (*D*) by

4 MSD(Δt)=4DΔt

The *MSD* is then corrected for localization uncertainty and blur (see Supporting Information). We also performed an accuracy study for a wide variety of trajectory analysis methods (e.g., single step (SS), all-pairs (AP), independent-pairs, regression-based, trajectory based, *MSD* scaling, and cumulative squared displacement). These methods are variably susceptible to linking errors when used with cost-matrix minimization algorithms and the particle concentration is elevated. Our findings indicate that the SS methodology, which computes an average diffusion constant for a trajectory based on position differences between consecutive frames, is among the best at resisting bias from linking errors (see Supporting Information for details). Single-step analysis is the primary method used here.

### Particle Surface Concentration

The circular illumination area was approximately 37 μm in diameter, with an area of ∼ 1100 μm^2^. Particle surface concentration was computed as a spatial average over the measured illuminated area. However, as documented in the Supporting Information, the ability to distinguish unique fluorescent spots increases nonlinearly as a function of particle density and is also diffusion constant dependent. For our set up, nonlinearities impact lipid surface concentration measurements above ∼ 0.05 particles μm^–2^).

### Diffusion Simulator

Computer simulations enable direct comparison to experimental results and provide additional assessment of measurement accuracy. Details of the basic diffusion simulation algorithm have been published previously.^29^ We adapted this algorithm to simulate 2D Brownian motion of lipid molecules within a bilayer suspended on a MECAopto-inv chip, replicating the wide-field optical conditions produced by our dual-mode microscope. The wide-field laser illumination was approximated using a circularly masked 2D-Gaussian intensity profile. By adjusting both the mask diameter and Gaussian width, the experimentally observed laser illumination profile was closely mimicked. Fluorescent lipids were modeled as point particles with a photon emission distribution defined by a Gaussian point spread function (PSF) scaled to match observations in our system (∼280 nm radius). A more detailed discussion of the PSF is included in the Supporting Information. The rate of emission from simulated molecules was proportional to the intensity of illumination. The total number of photons emitted by a single molecule before photobleaching was determined by sampling from an exponential distribution based on the observed limits of the fluorescent lipid (Cy5-DSPE) used in this study. Other simulation input parameters included the camera resolution, detector noise levels, fluorescent particle emission rate, the particle surface density, the 2D diffusion constant, the laser illumination time, and the camera frame interval. To produce data that closely matched experimental observations, background counts were combined with simulated fluorescent signal from point emitters. Each simulated frame was then compiled into a video (or image stack) that could be processed by PT or TM algorithms in an identical manner to experimentally acquired data. A detailed comparison of the correspondence between the simulator output and experimentally acquired data is provided in the Supporting Information.

## Results and Discussion

### Trajectory Formation

We selected bilayers for trajectory analysis that formed flat membranes by qualitatively evaluating the size of fluorescent spots in the camera images. Bilayer curvature produces significantly defocused spots over a portion of the illuminated area, often near the edges. Significant curvature was apparent for ∼ 10–20% of the bilayers. These bilayers were ignored. Figure 1D shows an example image from a flat membrane with fluorescent spots and overlaid trajectories defined by either TM or PT SPT algorithms. Additional raw images, including a video typical of the membranes formed, can be found in the Supporting Information.

Like most SPT algorithms, both TM and PT preprocess all frames in the time sequence to enhance spatial features and reduce noise. Particles are detected using intensity-based thresholding or other segmentation techniques. Additionally, TM and PT link the movement of particles across time frames into meaningful trajectories by identifying all the nearest-neighboring spots in a subsequent frame and optimizing a cost matrix that includes variables such as displacement, particle intensity, and expected motion type. The optimization process identifies a corresponding spot that most likely arises from the same molecule. Because cost-matrix minimization is inherently probabilistic, the trajectory formation algorithm is inherently susceptible to error.

SPT methods typically also require users to specify a maximum displacement value for particles as well as a time-based link range. We refer to the displacement value as the maximum link radius (MLR), which is the maximum radial distance over which the algorithm considers possible linkages in a subsequent frame. In addition, a time-based link range setting allows bridging of potential discontinuities in a trajectory due to fluorescent blinking, or stochastic intensity fluctuations in the molecule. We examined numerous single-molecule trajectories in our freestanding bilayers at low fluorescent particle concentration within the membrane (i.e., ∼ 0.02 particles μm^–2^). In this range, the integrity of individual trajectories could be validated by eye and we found no evidence of blinking or intermittent dimming over time. Thus, we set the time-based link range equal to one frame for all analyses (i.e., we assumed no dark gaps in a trajectory). However, we changed the displacement value systematically in order to find the optimal MLR. Identifying the proper MLR for microelectrode cavity arrays via a dual-modality optical system is the key method developed in this study and is necessary for accurate determination of diffusion constants.

### FCS Measurements

In order to ensure the proper MLR for a given optical configuration and bilayer sample, we acquired confocal-FCS measurements at various locations and at different laser powers by collocating the beam waist in the lipid bilayer. Because fluorescent data are collected from a diffraction-limited focal point, signal distortions due to bilayer geometry, fluorescent particle density, and background autofluorescence from the chip materials are minimized. Performing the measurement at slightly different locations ensures homogeneity and reproducibility. Performing measurements at different optical powers ensures that photobleaching does not bias the diffusion constant measurements. Figure 2 shows representative autocorrelation curves from three separate measurements in the same bilayer. Fitting the curves collectively produces a diffusion constant of 11 ± 2 μm^2^ s^–1^. This result is used to establish optimal SPT settings.

**Figure 2:**
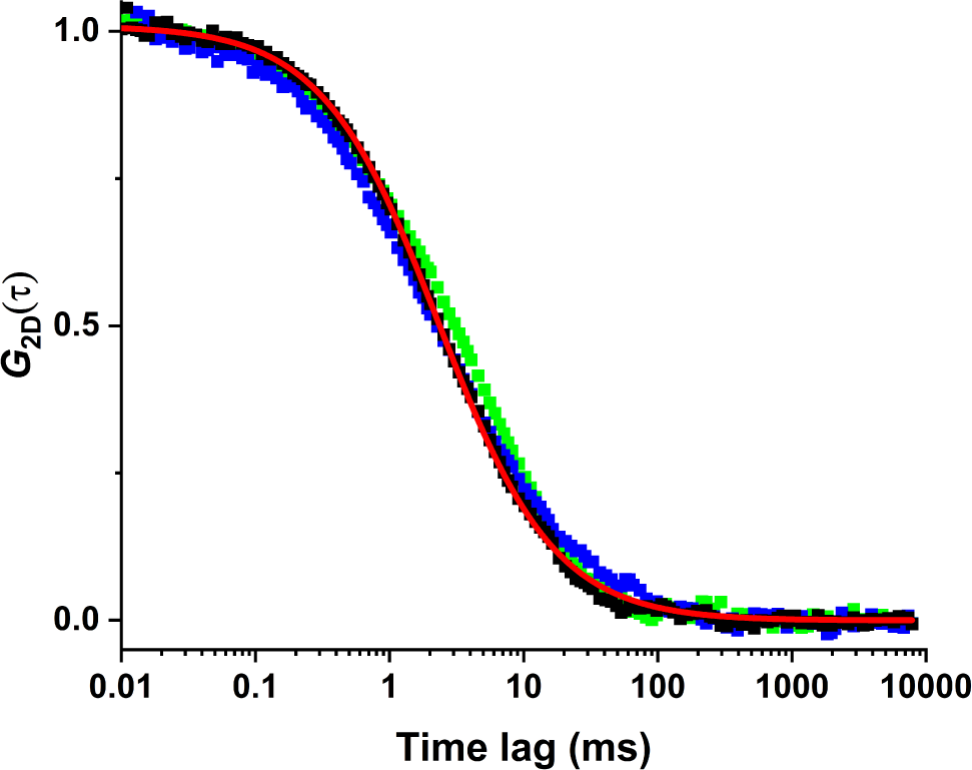
Autocorrelation curves of photon bursts from Cy5-DSPE acquired in the confocal mode from the same bilayer at optical powers of 5.3 kW/cm^2^ (black), 21 kW/cm^2^ (blue), 60 kW/cm^2^ (green) and three different positions. Fitting the data with eq 1 (red) yields a diffusion constant of 11 ± 2 μm^2^ s^–1^.

### Calibrating the MLR with FCS

At the particle densities desired for most ion channel and membrane protein experiments in microelectrode cavity arrays (0.01–0.1 particles μm^–2^), SPT algorithms can introduce significant errors into the calculated diffusion constant if the particle processing algorithm is not appropriately calibrated. Using an MLR that is too low underestimates the diffusion constant by linking spots into short, fragmented trajectories. If the MLR is set too high, the diffusion constant is overestimated because false links are created between particles at unrealistically large distances. The impact of a nonoptimal MLR on the diffusion constant is illustrated in Figure 3, where an ∼ 5-fold change in the MLR (from 1.7 to 8.4 μm) produces an ∼ 7-fold shift (10^0.85^ – 10^1.7^ μm^2^ s^–1^) in the centroid of blur-corrected diffusion constant distributions, as detailed in Figure 3A and B.

**Figure 3:**
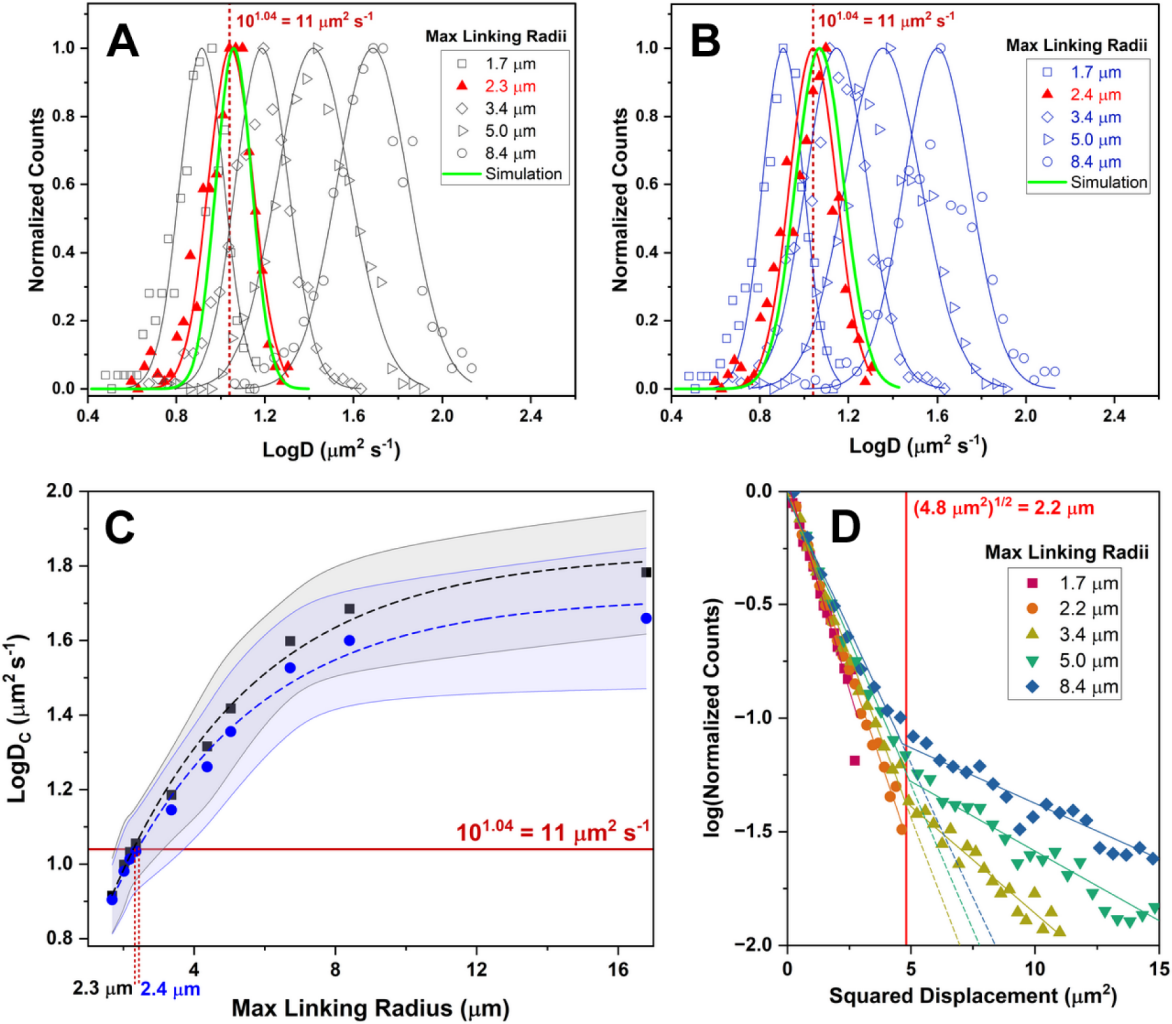
Trajectory-based diffusion constant distributions at ∼0.035 particle μm^−2^ from Particle Tracker for (A) single-step (SS) and (B) all pairs (AP) nonregression MSD analysis fit to a logarithmic Gaussian. The distributions shift to higher values of D due to linking errors. Red curves correspond to trajectories analyzed with the optimal MLRs (2.3 and 2.4 μm, respectively). Green curves correspond to Brownian diffusion simulations performed at 11.0 μm^2^ s^–1^. (C) Summary of the diffusion constant distribution centroids (D_c_) as a function of the MLR from 3A (black squares) and 3B (blue circles). Shaded areas indicate ± 1 standard deviation. The diffusion constant determined by FCS (11 μm^2^ s^–1^, red line) is used to establish the optimal MLR (e.g., 2.3 to 2.4 μm, respectively) for SPT analysis. (D) Squared displacement histograms for 5 different MLRs: 1.7 μm (red), 2.2 μm (orange), 3.4 μm (yellow), 5.0 μm (green), and 8.4 μm (blue). The location of the kink in the histogram approximates the optimal MLR (2.2 μm, red), which agrees well with the MLR determined using FCS.

Wieser and Schütz^30^ developed a method for determining the optimum linking radius using simulated data and plots of diffusion constant vs search radius. The method did not involve cost-matrix minimization, as in PT or TM, but instead linked individual particles in a nearest-neighbor fashion using a prescribed radial distance from the spot’s original location. Their graphical analysis showed that the diffusion constant reached a plateau, and the point where this occurred was used to determine the optimal radius. The method performed well for surface densities up to ∼ 1 particle μm^–2^ and a diffusion constant of 1 μm^2^ s^–1^.

In effort to find an optimal MLR for PT and TM algorithms using data from freestanding bilayers, we applied a similar approach by plotting the centroid fit of the diffusion constant distribution (plotted as logD_c_) from SS and AP analyses as a function of MLR. As can be seen in Figure 3C, our data also show a distinct plateau at large MLRs, similar to Wieser and Schütz.

However, unlike the method of Weiser and Schütz, the onset of the plateau does not correspond to a diffusion constant that agrees with FCS. Figure 3C shows that the onset of the plateau in logD_c_ occurs at an MLR of ∼8 μm. When this MLR is utilized by SPT algorithms, an erroneously large diffusion constant results (∼60 μm^2^ s^–1^, or logD_c_ ≈ 1.7 μm^2^ s^–1^, see Figure 3A and B). The discrepancy is apparently a consequence of the experimental conditions necessary for detecting single lipid molecules. In our case, the values of *D* are ∼10x larger and the necessary camera exposure time is long (Δ*T*_exp_ = 40 ms). Even though the label concentration is low (0.035 particles μm^–2^) in comparison to Weiser and Schütz, these factors render the plateau method ineffective for optimizing the MLR.

To observe a plateau in logD_c_ at the correct MLR value, the particle density must be low enough so that the closest distance between nonrelated particles is generally greater than the MLR. When the particle density grows too high (for a given *D* and Δ*T*_exp_), nonrelated particles can be linked together with an incorrectly large displacement. This creates trajectories that overestimate the true diffusion constant. Therefore, using the method of Weiser and Schütz to determine the optimal MLR is unsuitable for many experimental scenarios relevant to microelectrode cavity arrays, especially those targeting ion-channel proteins, which often occupy the bilayer surface at densities exceeding 0.03 particles μm^–2^.

Since FCS uses an ensemble average to determine spatially localized diffusion constants in a manner that is not dependent on the linkage of individual particles, FCS provides a more robust way to select the proper MLR for SPT. Using the value for *D* determined from FCS to estimate the appropriate MLR (Figure 3C, red lines) gives a much smaller value (∼2.3 to 2.4 μm) than the plateau method. These smaller MLRs correspond to the diffusion constant distributions shown in Figure 3A and B (highlighted in red) with centroids that correspond to the value determined by FCS analysis. Selecting the MLR by employing FCS calibration generates more accurate SPT results, even under elevated fluorescent particle density, large values of D, and long Δ*T*_exp_.

To further explore the impact of erroneous linkage, we applied the theory of Kerkhoff and Block.^31^ Their approach to error detection uses histograms of SDs between consecutive frames, plotted on a semilogarithmic scale. For accurately linked trajectories undergoing homogeneous Brownian motion, these histograms should appear linear. Deviations from linearity indicate tracking errors. They explored the theory using particle simulations with varying diffusion constants (0.1–1.0 μm^2^ s^–1^), particle densities (0.02–0.27 particles μm^–1^), and different trajectory linking algorithms. We applied the theory to measurements in microelectrode cavity arrays at ∼ 0.035 particles μm^–2^. Figure 3D shows deviations from linearity at both small and large MLRs. MLRs that are too small cause a slight negative deviation (red). MLRs that are too large cause positive deviations (olive, green, blue). Fitting these data to a piecewise linear function allows an SD threshold to be identified at the location of common discontinuity. This threshold can be interpreted as the optimal MLR, which we estimate to be 2.2 μm. This value agrees with the optimal MLR determined by calibrating the SPT algorithm with FCS diffusion results (Figure 3C) and validates the FCS calibration methodology.

We also compared the MLR-optimized SPT lipid diffusion constants to output from a 2D Brownian motion simulator. Figure 3A (green) and B (green) show ground-truth results from simulations where the specified diffusion constant is exactly 11.0 μm^2^ s^–1^. As can be seen, excellent agreement between the simulation (green) and experiment (red) is achieved. This correspondence provides additional verification of the accuracy of FCS-calibrated, blur-corrected tracking and diffusion constant calculations.

### Diffusion Coefficient Accuracy and Particle Density

To assess how surface concentration affects measurement accuracy, we conducted simulations that quantified the expected error as a function of particle concentration, maximum linking radius (MLR), and the known diffusion constant. These simulations modeled ideal Brownian motion without photobleaching across a range of diffusion constants, while varying surface particle density from 0.001 to 0.125 particles μm^–2^. After forming trajectories with TM, we then corrected the simulated output for both blur and localization uncertainty and computed a diffusion constant for each trajectory using the SS methodology. The distribution of diffusion constants for all simulated trajectories was characterized by fitting to a log-normal function (see Figure 3A). Simulations were repeated in triplicate to produce a mean and standard deviation for each set of conditions. Figure 4 presents the resulting diffusion estimates as a function of MLR and three input diffusion constants. The data show that substantial errors emerge when the MLR is not appropriately calibrated, even at moderately low particle densities (∼ 0.025–0.05 particles μm^–2^).

**Figure 4:**
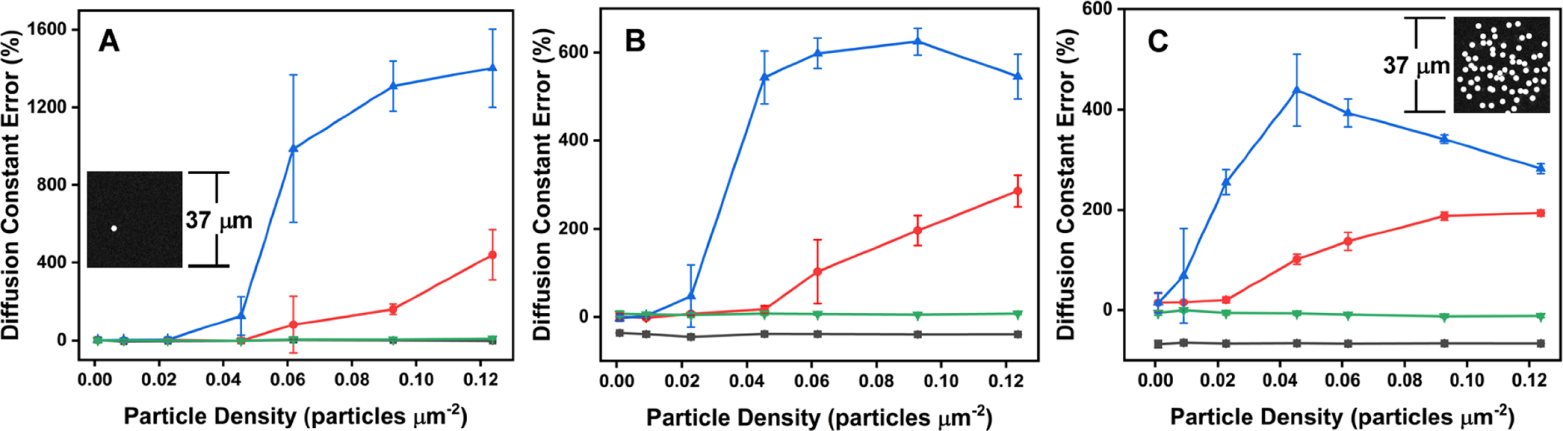
Diffusion constant error at (A) 1.0 μm^2^ s^–1^; (B) 5.0 μm^2^ s^–1^; (C) 11.0 μm^2^ s^–1^ processed with MLRs of 1.0 μm (black), 2.3 μm (green), 8.4 μm (red), 15.1 μm (blue). Positive errors arise from incorrect frame-to-frame particle linkages that have disproportionately large distances. Negative errors arise from incorrect exclusion of large distance links. The FCS-optimized MLR (2.3 μm, green) established at 11.0 μm^2^ s^–1^ remains essentially unaffected (<10% error) for all particle densities and simulated diffusion constants. Error bars indicate standard deviation (*N* = 3). Insets in (A) and (C) depict particle concetration and provide visual reference for the minimum and maximum particle densities of 0.001 and 0.125 particles μm^–2^, respecively.

High particle densities (>0.05 particles μm^–2^) cause an increased number of linking errors and correspondingly large errors in diffusion when the MLR is not optimized. MLRs above the optimum value of 2.3 μm (red, blue) produce positive deviations in the predicted diffusion constants at moderate particle density due to inclusion of too many long-distance linkages. MLRs below the optimum radius (black) incorrectly exclude a population of long-distance linkages, which creates a negative error (i.e., the predicted value of D is too small). Panels A, B, and C demonstrate that the sensitivity of deviation is also dependent on the magnitude of the simulated diffusion constant. Generally, when the MLR is not optimized (e.g., red, blue, black), SPT analysis for particles with a small diffusion constant can be performed more accurately than for particles with a large diffusion constant under relatively low-density conditions. High labeled particle density produces significant error for all diffusion constants determined with nonoptimal MLRs. However, an FCS-calibrated MLR of 2.3 μm (green) remains relatively impervious to error (<10%) over all tested surface densities and diffusion constants.

## Conclusions

We demonstrated successful single-chromophore detection and SPT methods within freestanding planar lipid bilayers formed on microelectrode cavity arrays. The commercially available chip positions the bilayer a convenient distance away from an underlying optical window, improves membrane stability, and enables simultaneous electrical characterization. When used with proper microscope optics, the background and spherical aberrations arising from the chip’s surface features do not prohibit high-sensitivity fluorescence detection within the bilayer. In this work, we performed single-chromophore tracking (i.e., Cy5-labeled lipids) over regions that were 37 μm in diameter (∼1100 μm^2^). We have also generated sufficient signal-to-background noise metrics to successfully track Cy5-labeled lipids across bilayer areas exceeding 75 μm in diameter (∼4400 μm^2^) using alternative illumination strategies.

We described a calibration method to minimize bias inherently present in particle trajectories formed by common SPT algorithms that employ cost-matrix minimization. Using a dual-mode illumination and detection strategy, we report a method for employing FCS to standardize SPT by determining an optimal MLR. Our studies show that a nonoptimal MLR causes significant bias in the resulting diffusion constant (e.g., −75% – 1300% error). Errors arise from either the formation of incorrect long-distance linkages (positive bias) or the improper exclusion of long-distance linkages (negative bias). The frequency of erroneous links within a trajectory is a function of the label surface density and the optical conditions necessary for detecting single chromophores.

Using an optimal MLR, we showed that error can be dramatically reduced (<10%) for a wide range of label concentrations (0.001–0.125 particles μm^–2^) and diffusion constants (1.0–11.0 μm^2^ s^–1^). Experiments in microelectrode cavity arrays aim to characterize adsorption, desorption, equilibrium dynamics, assembly kinetics, aggregation numbers, and multimodal lateral diffusion of various membrane proteins and ion channels. While such investigations can be performed under conditions that enable diffraction-limited localization of single molecules, they often require surface concentrations that are relatively high (i.e., not infinitely dilute). The dual-mode microscopy and calibration technique described here facilitates SPT and diffusion constant accuracy under surface coverages appropriate for many types of experiments.

Lastly, the calibrated and blur-corrected diffusion constant we determined for Cy5-DSPE in DPhPC lipid bilayers (i.e., 11.0 μm^2^ s^–1^) is consistent with previously reported values in similar freestanding bilayers.^9,10,15,32,33^ However, published values vary over a 4-fold range (i.e., ∼ 5–20 μm^2^ s^–1^).^15,33^ It is important to note that some of the reported values predate the development of relevant theories^30,31^ and, as a result, did not include efforts to optimize the search radius for specific experimental conditions. Given the magnitude of error that can occur as a function of MLR, a nonoptimized search radius, or a high particle concentration, could account for some of the apparent discrepancies. Additional confounding factors include the amount of solvent incorporated into the bilayer, the specific molecular structure of the lipids employed, or methods that neglect blur correction. Although absolute accuracy in SPT measurements is not always essential when experiments rely on relative changes in response to an experimental variable, calibration with FCS provides a universally reliable method. Furthermore, the general calibration strategy can generate benchmark diffusion measurements for model planar membranes of any type (e.g., freestanding, supported, and hybrid bilayers).

## Supplementary Material





## Notes

Description of the diffusion simulator, mathematical definitions, and trajectory correction methodologies, diffusion analysis accuracy comparison (PDF)
Video comparing single-molecule trajectories from simulated and experimental sources (MP4)

## References

[1] Shin J., Jeong S. H., Shon M. J. (2025). Advancing Membrane Biology: Single-Molecule
Approaches Meet Model Membrane Systems. BMB
Rep..

[2] Hartman K. L., Kim S., Kim K., Nam J.-M. (2015). Supported
Lipid Bilayers as Dynamic
Platforms for Tethered Particles. Nanoscale.

[3] Thompson J. R., Heron A. J., Santoso Y., Wallace M. I. (2007). Enhanced Stability
and Fluidity in Droplet on Hydrogel Bilayers for Measuring Membrane
Protein Diffusion. Nano Lett..

[4] Weatherill E. E., Wallace M. I. (2015). Combining
Single-Molecule Imaging and Single-Channel
Electrophysiology. J. Mol. Biol..

[5] Reina F., Galiani S., Shrestha D., Sezgin E., De Wit G., Cole D., Lagerholm B. C., Kukura P., Eggeling C. (2018). Complementary
Studies of Lipid Membrane Dynamics Using iSCAT and Super-Resolved
Fluorescence Correlation Spectroscopy. J. Phys.
Appl. Phys. D: Appl. Phys..

[6] Rose M., Hirmiz N., Moran-Mirabal J. M., Fradin C. (2015). Lipid Diffusion in
Supported Lipid Bilayers: A Comparison between Line-Scanning Fluorescence
Correlation Spectroscopy and Single-Particle Tracking. Membranes.

[7] McGuire H., Blunck R. (2022). Studying KcsA Channel Clustering
Using Single Channel
Voltage-Clamp Fluorescence Imaging. Front. Physiol..

[8] Heron A. J., Thompson J. R., Cronin B., Bayley H., Wallace M. I. (2009). Simultaneous
Measurement of Ionic Current and Fluorescence from Single Protein
Pores. J. Am. Chem. Soc..

[9] Ide T., Yanagida T. (1999). An Artificial Lipid
Bilayer Formed on an Agarose-Coated
Glass for Simultaneous Electrical and Optical Measurement of Single
Ion Channels. Biochem. Biophys. Res. Commun..

[10] Burden D. L., Kasianowicz J. J. (2000). Diffusion Bias and Photophysical Dynamics of Single
Molecules in Unsupported Lipid Bilayer Membranes Probed with Confocal
Microscopy. J. Phys. Chem. B.

[11] Borisenko V., Lougheed T., Hesse J., Füreder-Kitzmüller E., Fertig N., Behrends J. C., Woolley G. A., Schütz G. J. (2003). Simultaneous
Optical and Electrical Recording of Single Gramicidin Channels. Biophys. J..

[12] Harms G. S., Orr G., Montal M., Thrall B. D., Colson S. D., Lu H. P. (2003). Probing
Conformational Changes of Gramicidin Ion Channels by Single-Molecule
Patch-Clamp Fluorescence Microscopy. Biophys.
J..

[13] Chandler E. L., Smith A. L., Burden L. M., Kasianowicz J. J., Burden D. L. (2004). Membrane Surface Dynamics of DNA-Threaded Nanopores
Revealed by Simultaneous Single-Molecule Optical and Ensemble Electrical
Recording. Langmuir.

[14] Rajapaksha S. P., Wang X., Lu H. P. (2013). Suspended
Lipid Bilayer for Optical
and Electrical Measurements of Single Ion Channel Proteins. Anal. Chem..

[15] Pérez-Mitta G., Sezgin Y., Wang W., MacKinnon R. (2024). Freestanding
Bilayer Microscope for Single-Molecule Imaging of Membrane Proteins. Sci. Adv..

[16] Mueller P., Rudin D. O., Tien H. T., Wescott W. C. (1962). Reconstitution of
Cell Membrane Structure in Vitro and Its Transformation into an Excitable
System. Nature.

[17] Tsemperouli M., Amstad E., Sakai N., Matile S., Sugihara K. (2019). Black Lipid
Membranes: Challenges in Simultaneous Quantitative Characterization
by Electrophysiology and Fluorescence Microscopy. Langmuir.

[18] Ensslen T., Behrends J. C. (2022). A Chip-Based Array for High-Resolution Fluorescence
Characterization of Free-Standing Horizontal Lipid Membranes under
Voltage Clamp. Lab Chip.

[19] Burden D. L., Meyer J. J., Michael R. D., Anderson S. C., Burden H. M., Peña S. M., Leong-Fern K. J., Van Ye L. A., Meyer E. C., Keranen-Burden L. M. (2023). Confirming
Silent Translocation through Nanopores with
Simultaneous Single-Molecule Fluorescence and Single-Channel Electrical
Recordings. Anal. Chem..

[20] Jaqaman K., Loerke D., Mettlen M., Kuwata H., Grinstein S., Schmid S. L., Danuser G. (2008). Robust Single-Particle
Tracking in
Live-Cell Time-Lapse Sequences. Nat. Methods.

[21] Kowalek P., Loch-Olszewska H., Szwabiński J. (2019). Classification of Diffusion Modes
in Single-Particle Tracking Data: Feature-Based versus Deep-Learning
Approach. Phys. Rev. E.

[22] Maris J. J. E., Rabouw F. T., Weckhuysen B. M., Meirer F. (2022). Classification-Based
Motion Analysis of Single-Molecule Trajectories Using DiffusionLab. Sci. Rep..

[23] Lee B. H., Park H. Y. (2018). HybTrack: A Hybrid Single Particle
Tracking Software
Using Manual and Automatic Detection of Dim Signals. Sci. Rep..

[24] Reina F., Wigg J. M. A., Dmitrieva M., Vogler B., Lefebvre J., Rittscher J., Eggeling C. (2022). TRAIT2D: A Software for Quantitative
Analysis of Single Particle Diffusion Data. F1000Research.

[25] Simon F., Tinevez J.-Y., van Teeffelen S. (2023). ExTrack Characterizes
Transition
Kinetics and Diffusion in Noisy Single-Particle Tracks. J. Cell Biol..

[26] Simon F., Weiss L. E., van Teeffelen S. (2024). A Guide to
Single-Particle Tracking. Nat. Rev. Methods
Primer.

[27] Sbalzarini I. F., Koumoutsakos P. (2005). Feature Point Tracking and Trajectory Analysis for
Video Imaging in Cell Biology. J. Struct. Biol..

[28] Tinevez J.-Y., Perry N., Schindelin J., Hoopes G. M., Reynolds G. D., Laplantine E., Bednarek S. Y., Shorte S. L., Eliceiri K. W. (2017). TrackMate:
An Open and Extensible Platform for Single-Particle Tracking. Methods.

[29] Bailey D. J., Kindt J. T., Taylor M. M., Paulson A. R., Jones B. H., Hubbell K. L., Keranen-Burden L. M., Burden D. L. (2010). Post-Hoc Vibration
Mitigation for Single-Molecule Tracking and Diffusion Measurements
in Lipid Membranes. Spectrosc. Lett..

[30] Wieser S., Schütz G. J. (2008). Tracking Single Molecules in the
Live Cell Plasma Membrane-Do’s
and Don’t’s. Methods.

[31] Kerkhoff Y., Block S. (2020). Analysis and Refinement
of 2D Single-Particle Tracking Experiments. Biointerphases.

[32] Fahey P. F., Koppel D. E., Barak L. S., Wolf D. E., Elson E. L., Webb W. W. (1977). Lateral Diffusion in Planar Lipid Bilayers. Science.

[33] Sonnleitner A., Schütz G. J., Schmidt T. (1999). Free Brownian Motion of Individual
Lipid Molecules in Biomembranes. Biophys. J..

