# Supplementary material for: Accurate Single-Particle Tracking and Diffusion Measurement in Freestanding Lipid Bilayers and Model Membranes
Source: Anal Chem. 2025 Dec 8;97(50):27797–804. doi: 10.1021/acs.analchem.5c05037 (PMC12750401; doi:10.1021/acs.analchem.5c05037)
Supplement: Supplementary file 1 [file ac5c05037_si_001.pdf]

Supporting Information

Accurate Single Particle Tracking and Diffusion Measurement in Freestanding Lipid Bilayers and Model Membranes

Lily Anne Van Ye,[1] Richard D. Michael,[1] Joshua J. Meyer,[1] Sophia M. Peña,[1] Derek J. Bailey,[1] Lisa M. Keranen-Burden,[1] and Daniel L. Burden[1]

[1] Wheaton College, Dept. of Chemistry, Wheaton, IL 60187

Table of Contents

1. Diffusion Simulation

    a. Comparison of Simulated and Experimental Data S3

    b. Simulated Optical Background S5

    c. Spatially Averaged Fluorescent Spot Intensity Distribution S5

    d. Photobleaching S6

    e. Trajectory Length S7

    f. Particle Density Accuracy S8

2. Definitions, Corrections, and Accuracy Comparison

    a. Trajectory Analysis and Time Lag Type S8

    b. Correction for Localization Uncertainty and Blur S9

    c. Individual Trajectory Analysis via Single Step and All Pairs Methodology S10

    d. Ensemble All-Pairs Regression Analysis S10

    e. Trajectory-Based MSD Scaling Analysis S11

    f. Cumulative Squared-Displacement Analysis S11

    g. Comparison of Diffusion Analyses S11

    h. Summary of Diffusion Analysis Methodologies and Error Susceptibility S14

1. Diffusion Simulation

a. Comparison of Simulated and Experimental Data

Diffusion simulations were designed to closely mimic single-particle tracking data collected from MECAopto-inv chips and a microscope in the widefield mode using a 100x, NA = 1.49 objective. MECAopto-inv chips possess a ring-shaped Ag/AgCl electrode at the bottom of each microwell that, in combination with an iris, limit the size of the excitation laser beam on the suspended bilayer to an ~37 μm diameter circle (see Figure 1). Simulations also mimic the autofluorescence from the SU8 polymer layer in the chip, the photon collection efficiency of the optical system, and the various camera settings (e.g., illumination time, frame interval, gain, etc.). We qualitatively compared the similarity between the simulator output videos and experimental videos by visual inspection. Quantitative comparison was performed for the background count distribution, fluorescent spot intensity distribution, photobleaching rate, and trajectory length.

A detailed description of the core simulation engine has been published previously.[1] The basic algorithm simulates 2D Brownian motion of fluorescently labeled lipids in a bilayer by computing the distance moved (Δx) during a computational step time (Δt) according to

$$\Delta x = \sqrt{4D\Delta t}$$

where D is the diffusion coefficient. The direction of movement is defined by cardinal coordinates and determined by sampling from a uniform random number generator. Each simulated time step was 500 ns.

The simulated illumination profile was configured to approximate the microscope's illumination profile within the MECAopto-inv chips (~1100 μm^2) by employing a masked 2D Gaussian. A circular binary mask established the outer edge of the excitation region (~37 μm). A 2D Gaussian standard deviation (~11 μm) was used to control the gradient of the simulated illumination intensity across the mask. The emission rate from simulated fluorescent particles was defined by the particle's location within the illumination profile. Typically, the emission rate was set to a maximum value of 400 counts ms^{-1} at the center of the masked 2D Gaussian. Photocounts from simulated point particles were accumulated on a grid with a bin size equal to the calibrated camera pixel size of the optical system. To approximate the shape of the diffraction-limited fluorescent spots, simulated photocounts were locally dispersed based on random sampling from a 2D Gaussian point-spread function (PSF) with a 280 nm standard deviation. The centroid of the PSF aligned with the true (i.e., sub-pixel) location of the simulated particle at each time step.

For free-standing bilayer measurements, the EMCCD camera operated with a 40-ms exposure time and a 42-ms frame interval (23.8 Hz). The optical system's spatial resolution, calibrated using a USAF 1951 resolution grid, was 0.168 µm $pixel^{-1}$. To improve single-molecule signal collection, images were binned 2×2, yielding a final resolution of 0.336 µm $pixel^{-1}$. Simulated data were generated using the same exposure time, frame interval, and spatial resolution, with 0.168 µm $pixel^{-1}$ images also binned 2×2 to match the 0.336 µm $pixel^{-1}$ resolution.

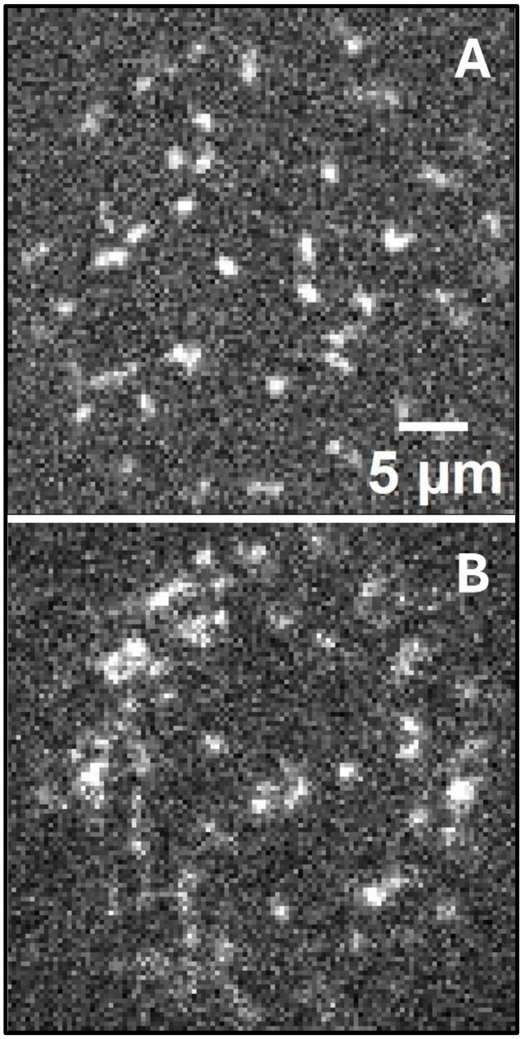

Figure S1: (A) Single frame image of simulated particles and (B) Cy5-DSPE lipids in a freestanding bilayer.

The density of spots, the illumination profile, the fluorescent spot emission intensity, the photobleaching of individual spots, and the diffusion constant were all controllable parameters within the simulation. Each parameter was adjusted to align with experimental conditions. A representative image of diffusing spots from a measurement of single Cy5-DSPE lipids in a freestanding DPhPC bilayer is shown in Figure S1. As can be seen, the image from the simulator (Figure S1A) closely mimics the spot size, spot intensity, surface coverage, and the circular illumination profile observed in experiments (Figure S1B).

In order to match the blurred appearance of diffusing lipids in MECAopto-inv-supported bilayers, a simulated PSF of 280 nm was required. This was larger than the measured PSF for stationary Cy5 molecules on bare glass (i.e., 160 nm). The latter PSF arises from the diffraction limit of the microscope's optical system using an ideal optical interface. We attribute the extra broadening required to match the simulated spot size to aberrations introduced by the MECAopto-inv chip. High-NA objectives (e.g., NA = 1.49) collect fluorescence photons from a suspended bilayer using a large solid angle that is complex. Some photons arrive at the detector after crossing the vertical side walls of the polymeric SU8, outside the Ag/AgCl electrode ring, and through the chip's bottom cover glass. Because these high-angle optical paths pass through multiple interfaces with mismatched refractive indices, the ideal PSF is degraded. Furthermore, the electrode ring partially obstructs the collection of fluorescence photons. Together, these factors introduce distortions absent from PSF measurements of single dye molecules on a cover glass. However, these non-idealities do

not degrade the signal quality enough to prohibit detection of individual chromophores. Fluorescent spots from individual molecules within the freestanding bilayer can be clearly distinguished.

Video S1 shows both simulated (A) and experimental (B) trajectories from fluorescent spots in the circular illumination area evolving over time (12.6 s) in 42-ms intervals at 0.035 particles μm^{-2}. As is visually evident, the simulator produces strong qualitative similarity to the experiment after processing with TrackMate single-particle tracking (SPT) software. As described in the main text, the simulated and idealized homogeneous Brownian motion closely approximates the diffusion of labeled lipids in freestanding bilayers.

b. Simulated Optical Background

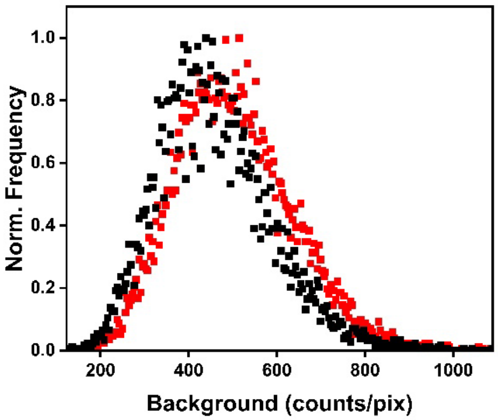

Figure S2: Background pixel intensity distributions from images binned 2x2: simulations (red), MECAopto-inv chip (black). Simulated background closely mimics the background observed in experiments.

To estimate the widefield background levels produced by scattering and autofluorescence in the MECAopto-inv chip, the number of background counts per pixel from 64 separate regions devoid of fluorescent spots (total of 12,544 pixels) were analyzed in histogram format (See Figure S2). Simulated background counts were added to each grid location by sampling randomly from a Gaussian distribution with a small exponential tail. The mean and standard deviation of the simulated background distribution, along with the duration and amplitude of the exponential tail, were adjusted to replicate the distribution observed in our apparatus. Using this approach, simulated background represents the sum total of camera read out noise, autofluorescence from the MECAopto-inv chip, and background associated with Raman and Rayleigh scattering. Figure S2 shows example background distributions from simulated and experimental images that are closely matched.

c. Spatially Averaged Fluorescent Spot Intensity Distribution

We evaluated single-particle tracking performance using both the Particle Tracker and TrackMate plugins in Fiji. TrackMate computes and reports the spatially averaged intensity of pixels within a detected spot. Thus, simulated trajectories were calibrated to replicate the spot intensity distributions observed in the TrackMate output derived from experimental videos. Eight user-

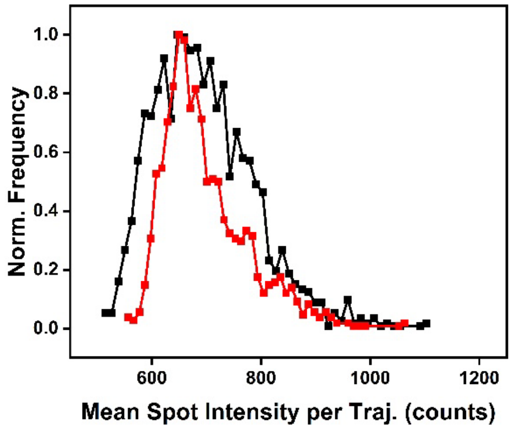

Figure S3: Mean spot intensity distributions from simulated (red) and experimental (black) data sets. The mean is defined as the spatially averaged spot intensity for all spots linked within a single trajectory, including spots that travel a large distance across the illumination area. Other simulation parameters were adjusted to match experimental conditions with D = 11 $\mu m^2 s^{-1}$.

defined parameters influence the simulated mean spot intensity: (1) simulated background levels, (2) PSF width (280 nm), (3) peak intensity at the PSF center, (4) illumination intensity profile, (5) diffusion constant, (6) frame exposure time, (7) photobleaching yield, and (8) particle density. Because spot intensity depends on both particle motion across the ~1,100 μm² illumination area and motion-induced blur, we averaged TrackMate's spot intensity output over time for each trajectory (Figure S3). Simulations were adjusted to mimic experiments, where the averaged spot intensity per trajectory produced ~650 counts (without background subtraction). The broader distribution observed from MECAopto-inv chips likely reflects a non-ideal Gaussian illumination profile arising from subtle laser interference patterns. These spatial features were not replicated in simulations but do cause increased emission intensity variations for molecules in bilayer experiments as they diffuse across the illuminated area.

d. Photobleaching

Simulated photobleaching was implemented by assigning each particle a photon yield drawn from an exponential distribution that was calibrated to match experimental values for Cy5-DSPE. The maximum emission rate was set to 400 counts ms^{-1}. As photons were emitted, each particle's photon budget was decremented until it reached zero, at which point the particle was removed and replaced by a new particle positioned outside the observation region. This approach maintained a constant total number of simulated particles, but resulted in a decreasing particle density at the center of the illumination area as time elapsed. Consequently, as evident in Video S1, the impact of photobleaching is

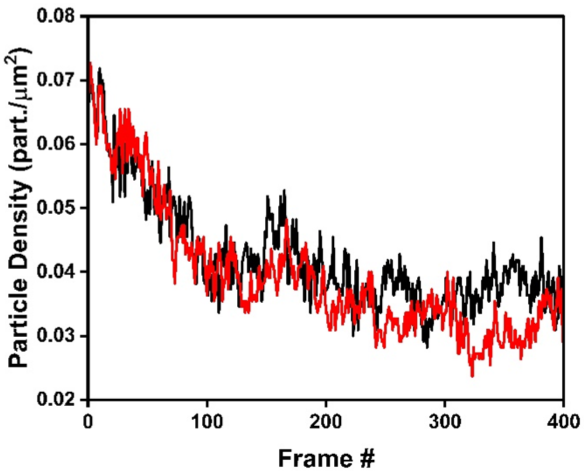

Figure S4: Photobleaching reduces the fluorescent particle surface density until equilibrium is reached. The photobleaching rate for simulations (red) was adjusted to match the photobleaching rate observed for bilayers containing Cy5-DSPE (black). The time interval was 42 ms $frame^{-1}$.

increasingly apparent in the illuminated center over time, while the peripheral particle density remains largely unchanged.

To assess the simulator's accuracy in replicating photobleaching dynamics, we used TrackMate to quantify the total number of spots per frame over a 400-frame simulated video (16.8 s). These results were then compared to analogous videos of Cy5-DSPE diffusing in a lipid bilayer (see Figure S4). The simulated photon yield was set to 800,000 counts. At the illumination intensities typically used in our microscope, approximately 40% of particles photobleached within the first 100 frames (4.2 s). The particle density typically stabilized at ~60% of the initial value for the remainder of the video. As can be seen in Figure S4, the output from the simulations produced a good match to the experimental data.

All SPT and diffusion analyses of Cy5-DSPE in lipid bilayers were performed after photobleaching reached equilibrium. Specifically, only frames beyond the first 100 were analyzed. This excluded the initial period of rapid particle density change and is necessary to achieve accurate diffusion results because high particle density introduces artifacts in SPT-derived diffusion constants. By omitting early frames, the influence of changing particle concentration is reliably controlled.

e. Trajectory Length

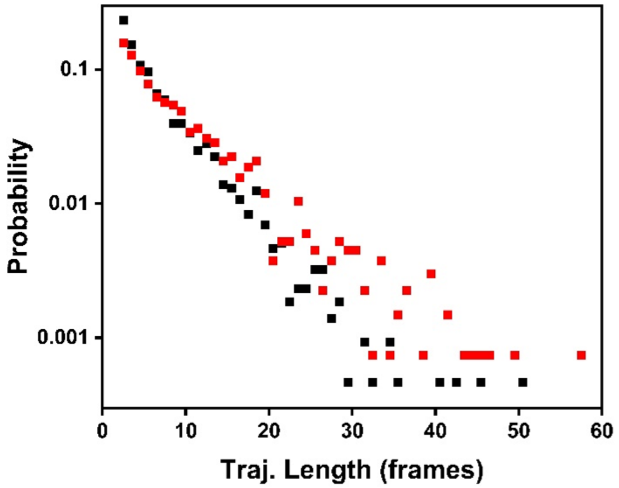

Figure S5: Trajectory length distribution of Cy5-labled lipid (black) and simulated particles (red) are similar.

Both Particle Tracker and TrackMate link spots into trajectories over the illuminated area of the bilayer. If the full set of empirically adjusted simulated conditions faithfully mimics the optical conditions established for MECAopto-inv chips, the distribution of trajectory lengths for both experimental and simulated data sets should be similar. To compare fluorescent spots from the two sets, we used TrackMate to form trajectories using identical values for object diameter, maximum linking radius (MLR), and quality threshold. Only trajectories formed after the video recordings reached photobleaching equilibrium were included. As can be seen in Figure S5, a good match between simulated and experimental trajectory length distributions was observed.

f. Particle Density Accuracy

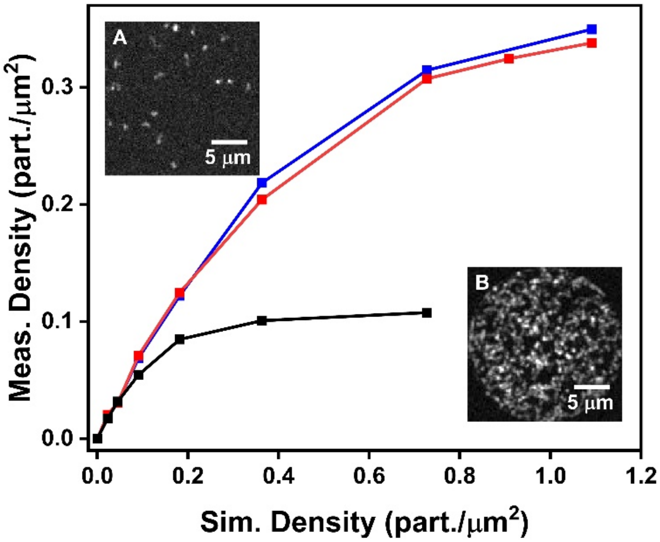

Figure S6: As the particle density increases, the measured particle density determined by TrackMate becomes less accurate. Particles with large diffusion constants are subject to more error than smaller diffusion constants. Black: D = 11 $\mu m^2\ s^{-1}$; Red: D= 1 $\mu m^2\ s^{-1}$; Blue: D = 0.1 $\mu m^2\ s^{-1}$ (resolution = 0.336 μm/pixels). Insets (A) and (B) provide visual reference for particle densities of 0.02 and 0.73 particles μm^{-2} (D= 11 $\mu m^2\ s^{-1}$), respectively.

Particle densities are determined by counting the number of fluorescent spots within an image and dividing by the illuminated area within the frame. However, the ability of SPT algorithms to distinguish unique spot locations is dependent on several factors, including surface crowding, the diffusion constant, the camera exposure time, and the signal-to-noise ratio. To assess the accuracy of particle density measurements, we controlled the particle density via simulation and compared it to measured values produced by Particle Tracker and TrackMate SPT algorithms. Figure S6 shows typical results. As can be seen, measured particle densities closely match the simulated values at low concentration. However, the accuracy of the SPT algorithm decreases dramatically as the surface concentration grows. Particles with large diffusion constants induce greater inaccuracy (i.e., non-linear deviations and saturation). The insets of Figure S6 provide visual reference for particle densities at the tested high- and low-density extremes (A = 0.02 particles μm^{-2}; B = 0.73 particles μm^{-2}) for a diffusion constant of 11 $\mu m^2\ s^{-1}$. A practical upper limit for accurate surface concentration measurement of single lipids in our system is ~0.05 particles μm^{-2}.

2. Comparing Trajectory Analyses for Accuracy

a. Trajectory Analysis and Time Lag Type

We compared diffusion results from different types of mean squared displacement (*MSD*) analyses (e.g., non-regression, regression, ensemble averaged, and single trajectory) as well as cumulative squared displacement (CSD) methods. For *MSD* processing, important distinctions between types of time lag are highlighted. Single-step (SS) analysis computes the squared displacement (SD) of individual particles between consecutive frames (i.e., a time lag of 1 frame)[2] and reports the mean within an individual trajectory. All-pairs (AP) analysis uses an iterative step-wise increase in the time lag to compute SDs and accounts for every possible pair of time points

within a trajectory. The time lag is limited to 10-15% of the trajectory length to minimize systemic bias.[2] Unlike the independent pairs (IP) method, which considers consecutive step-wise increases in time lag, the AP method leverages all available time intervals without neglecting information. However, the AP method creates a mutual dependency between trajectory points, especially at large lag times, which can bias results when time lags are large.

Assuming the unsupported bilayer is planar, the *MSD* for each time lag (Δt) is computed as

$$MSD(\Delta t) = \langle SD(\Delta t) \rangle = \langle r^2(\Delta t) \rangle = \langle (x(t+\Delta t) - x(t))^2 + (y(t+\Delta t) - y(t))^2 \rangle \quad (1)$$

and is related to the diffusion constant (D) by

$$MSD(\Delta t) = 4D\Delta t^{\alpha} \quad (2)$$

where the anomalous diffusion exponent (α) is equal to a value of one for pure Brownian motion.

b. Correction for Localization Uncertainty and Blur

This straightforward definition of MSD adequately describes diffusion constants for particles when there is no uncertainty in the particle position (i.e., localization error).[2,3] However, all optical systems suffer from the inability to determine the exact spatial location of single molecules. Even when particles are completely stationary, statistical noise from the camera readout obscures position information. Furthermore, the Brownian motion of fluorescent particles in the bilayer during the camera exposure introduces additional bias in the form of blurring. We compensated for both localization and motion blurring errors by adding correction factors to eq. 2. Localization errors were addressed by introducing a correction term of $4\sigma_{pos}^2$, where σ_{pos}^2 describes the spatial variance of a stationary particle.[4] In comparison to distances typically traveled by single molecules in a lipid bilayer in one frame, the instrument localization uncertainty in our instrument is small (i.e., σ_{pos}^2 = 0.007 μm^2), as determined by evaluating adherent dye molecules on a cover glass. Motion blurring can be corrected using a blur coefficient, R.[5–8] For homogeneous illumination during an exposure period of $\Delta T_{exp} \leq \Delta T$, R is given by

$$R = \Delta T_{exp}/(6\Delta T) \quad (3)$$

which simplifies to 1/6 for cases when the exposure time (ΔT_{exp}) equals the recording time interval between frames (ΔT). The resulting bias in *MSD* for simple Brownian motion is represented by:[8,9]

$$MSD(\Delta t) = 4D\Delta T\left(N_p - 2R\right) + 4\sigma_{pos}^2 \quad (4)$$

where $N_p$is an index in time lag units and $\Delta t = N_p \Delta T$. As can be seen, blur correction becomes less significant at large time lag indices because the average distance traveled by a diffusing species is generally large relative to the size of blur and the localization uncertainty. Blur correction is also less significant if $\Delta T_{exp} \ll \Delta T$. Eq. 4 predicts that full exposure ($\Delta T_{exp} = \Delta T$) underestimates the *MSD* by nearly 1/3 for small N_p.

c. Individual Trajectory Analysis via Single Step and All Pairs Methodology

Single-step (SS) diffusion analysis uses a corrected *MSD* (MSD_{corr}) computed between consecutive frames ($N_p = 1$) to yield a blur-corrected diffusion constant, D_{corr}, characteristic of the entire trajectory. The general D_{corr} associated with individual trajectories, with $\Delta t = N_p \Delta T$, is calculated by:

$$D_{corr} = \frac{N_p\left[MSD(\Delta t) - 4\sigma_{pos}^2\right]}{4\Delta t\left(N_p - 2R\right)} = MSD_{corr}(\Delta t)/(4\Delta t) \tag{5}$$

where Δt is the total time lag between frames. All-pairs diffusion analysis also uses eq. 5., but takes every possible pair of points in the entire trajectory and computes D_{corr,N_p} ($N_p = 1\ to\ N_{p,max} = 0.10N$, where N is the total number of points in the trajectory). Independent-pairs analysis is computed similarly, but uses consecutive non-overlapping pairs of points. The average diffusion constant for the trajectory ($\overline{D}_{corr}$) is then computed by weighting each D_{corr,N_p} by either the integer truncation of lag times, or the inverse of the variance computed for D_{corr,N_p} (σ_D^2):

$$\overline{D}_{corr} = \frac{\sum_{i=1}^{Np,max}(\lfloor N/N_p \rfloor)_i D_{corr,i}}{\sum_{i=1}^{Np,max}(\lfloor N/N_p \rfloor)_i} \tag{6}$$

$$\overline{D}_{corr} = \frac{\sum_{i=1}^{Np,max}(1/\sigma_D^2)_i D_{corr,i}}{\sum_{i=1}^{Np,max}(1/\sigma_D^2)_i} \tag{7}$$

d. Ensemble All-Pairs Regression Analysis

All pairs regression fits $MSD_{corr}(\Delta t)$ vs. Δt to a line (eq. 2, assuming α = 1) after conducting stepwise increases in the time lag (Δt). The slope of the line equals $4D_{corr}$. Using this approach, deviations from linearity can reveal the presence of anomalous diffusion (i.e., $\alpha \neq 1$). One variation takes lag data from all diffusing particles together and computes an ensemble average of displacement for each time lag to give a reliable value for D_{corr}. To avoid inducing errors,[2,8] the time lag (Δt) does not exceed 10% of the total trajectory length.

$$\Delta t_{max} = 0.10 \cdot N \cdot \Delta T \tag{8}$$

e. Trajectory-Based MSD Scaling Analysis

When $\alpha \neq 1$, linearizing eq. 2 transforms the anomalous diffusion exponent (α) to a slope and the blur-corrected diffusion constant (D_{corr}) to an intercept:

$$\ln[MSD_{corr}(\Delta t)] = \alpha \ln(\Delta t) + \ln 4D_{corr} \tag{9}$$

Our implementation uses AP processing of individual trajectories up to $\Delta t_{max} = 0.15 \cdot N \cdot \Delta T$ to yield a distribution of D_{corr} and α values.

f. Cumulative Squared-Displacement Analysis

The cumulative squared displacement (CSD) distribution, $C(r_{corr}^2, \Delta t)$,[10,11] was constructed by correcting $r^2(\Delta t)$ for blur and localization uncertainty,

$$r_{corr}^2(\Delta t) = \frac{N_p[r^2(\Delta t) - 4\sigma_{pos}^2]}{(N_p - 2R)} \tag{10}$$

sorting $r_{corr}^2(\Delta t)$ values in ascending order and assigning ranks to each data point. The distribution function was then defined as

$$C(r_{corr,j}^2) = 1 - j/N \tag{11}$$

where j is the rank of a given data point in the sorted order and N is the total number of data points. For homogeneous 2D Brownian motion, the expected form of the CSD distribution follows

$$C(r_{corr,j}^2, \Delta t) = e^{-r_{corr}^2 / 4D_{corr}\Delta t} \tag{12}$$

CSD analysis can identify trends in direction persistence or confinement that MSD analysis may overlook.

g. Comparison of Diffusion Analyses

Numerous methods for measuring diffusion via particle tracking methods exist. Here, we apply the FCS calibration method to a few common regression-based approaches. Analyzing displacement as a function of time lag provides time-resolved information obscured by SS or AP single-trajectory averaging (i.e., eqs. 5, 6, and 7). Plots of *MSD* versus time lag reveal the presence of different diffusion modes, such as normal Brownian motion, subdiffusion, or superdiffusion. Additionally, when the molecular environment is complex, regression-based analyses can provide size estimates of confinement domains or corrals that influence particle motion.

Figure S7A shows an AP *MSD* regression analysis used to determine the lipid diffusion constant. Each time lag for all trajectories is averaged as an ensemble. After calibration with FCS to determine the proper MLR and blur correction, the *MSD* for all time lags up to 10% of the maximum trajectory length gives a diffusion constant that exactly matches FCS observations. The inset to Figure S7A shows a trajectory length histogram for all detected trajectories. Only trajectories longer than 10 frames (dark bars) are included in the analysis. Altering the trajectory-length filter to 4 frames with a 25% time-lag limit produces negligible differences in the value of the resulting diffusion constant.

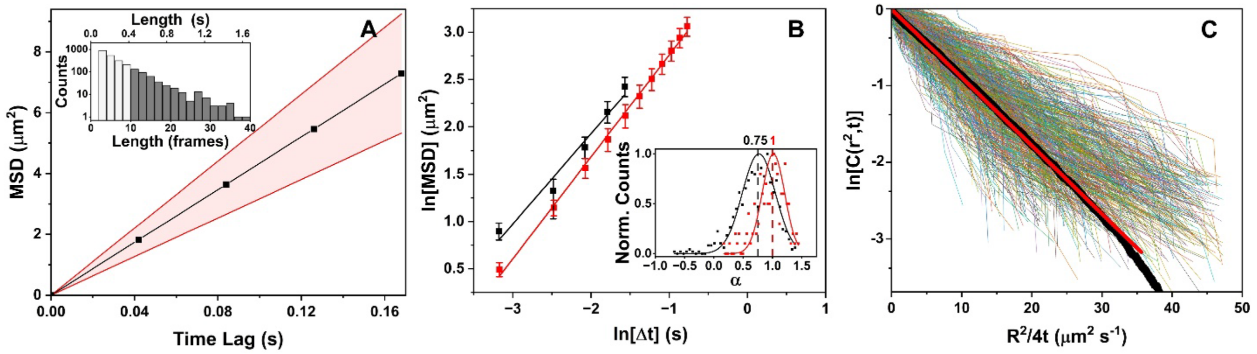

Figure S7: *(A) Ensemble all-pairs (AP) trajectory analysis with time-lag linear regression. MSD average of 413 trajectories (±1 standard deviation, pink) with an MLR of 2.3 μm yields a diffusion constant of 11.0 ± 0.1 μm² s⁻¹, matching the value produced by FCS. (A, inset) Trajectory length distribution, N=2543 trajectories from a 16.4 s video. (B) MSD scaling analysis for two individual trajectories. Simulated Brownian motion (red) and DHPE-Cy5 lipids (black) give diffusion constants of 11.4 ± 1.7 μm² s⁻¹ and 11.5 ± 0.4 μm² s⁻¹, respectively. Errors reported as SEM. (Inset) The anomalous diffusion exponent distribution (black) suggests constrained lipid motion (α=0.75). However, further investigation reveals this arises from a common SPT algorithm artifact (see text). (C) CSD analysis of individual (fine) and averaged trajectories (bold black) gives a homogeneous diffusion constant of 11.25 ± 0.01 μm² s⁻¹ (red line). All three approaches (A, B, C) give similar diffusion constant values following MLR calibration and blur correction.*

Figure S7B presents an MSD scaling analysis for two individual trajectories. In this plot, lag times (Δt) are computed within single trajectories (i.e., not averaged over all trajectories). This approach preserves rare or outlier behavior and can detect heterogeneity in molecular diffusion that would otherwise be masked in an ensemble average. Blur-corrected data from lipid diffusion experiments (black), along with a simulated dataset (red) are analyzed via SPT (using the calibrated MLR of 2.3 μm) and were fit using eq. 9. The simulation aimed to replicate the experimentally measured particle density (~0.035 particles μm^{-2}), illumination area (i.e., 1100 μm²), and diffusion constant (11.0 μm^2 s^{-1}). Importantly, simulations were performed without photobleaching. Removing photobleaching creates trajectories that are significantly longer than the corresponding lipid data, as visually evident in Figure S7B by the larger lag times. However, the distribution of anomalous diffusion exponents for the lipid data (Figure S7B inset, α = 0.75) differs significantly from the simulated data (α = 1). This suggests labeled lipids in freestanding bilayers exhibit constrained motion, which is counterintuitive given the simplicity of the environment and presumed homogeneous Brownian motion.

Further investigation reveals the shift in the α distribution arises from a general artifact. The effect is serendipitously uncovered by the lack of photobleaching in the simulation, but is not caused by photobleaching. Instead, the shift is related to linking errors induced by common SPT algorithms with cost-matrix minimization. These probabilistic algorithms tend to select erroneously large displacements in favor of erroneously small displacements. For example, in addition to producing kinks in the SD histogram at large SDs (Figure 3D, main text), linking errors also bias the slope of the SD histograms at small SDs, *prior to the discontinuity*. In this region of the histogram, the linear slope grows progressively less negative as the MLR is increased. This is due to a more subtle influence of the erroneous large links (i.e., incorrect links cause more than just a large discontinuity). Output from simulations performed at near-infinite dilution (~0.001 particles μm^{-2}) where the SPT algorithm can only make correct links, do not change slope at small SDs (data not shown). Instead, the slope at small SDs remains constant for all MLRs. Thus, trajectories formed even at moderately low label concentration (e.g., ~0.035 particles μm^{-2}) contain bias from incorrect links. This finding indicates that as the label concentration goes up, the frequency of erroneously large linkages increases for a given MLR across all displacement scales. This effect is particularly relevant for measurements in microelectrode cavity arrays, where experiments must often be conducted at elevated surface concentrations. Consequently, errors in the calculated diffusion constant are expected to increase progressively with surface concentration.

Because all trajectories (i.e., both MLR-optimized and non-optimized) contain incorrect links, the *MSDs* computed at every time lag are influenced, even for trajectories defined using an optimized MLR. However, the influence is not uniform. Short time lags contribute more measurement points to the *MSD* than long time lags. In addition, the extra distance incorporated by the incorrect links represents a larger fraction of the total step distance at short time lags. Therefore, the impact of erroneously large links is preferentially greater for data points at short time lags. This subtly increases the computed *MSD* at short time lags and works to decrease the slope of the single-trajectory scaling analyses, as well as the y-intercept (which specifies *D*). Incorporation of incorrect large links systematically shifts the α distribution below one and produces negative bias in the centroid of the diffusion constant histograms computed by both AP and IP methods.

By excluding photobleaching in the simulation, numerous large time lags are processed due to the increased trajectory length. Long time lags are more resistant to linking errors and, when numerous enough, can overcome the statistical influence of data points at short time lags in the regression analysis. The result is a more accurate estimate of the anomalous diffusion exponent, which is shown in the inset for Figure S7B (red) to have a value equal to one. Diffusion constants computed by AP and IP regression-based methodology also become more accurate (i.e., they more closely match the simulation input parameter) when photobleaching is artificially excluded.

We confirmed this idea by including photobleaching in simulations and noting relevant shifts. As anticipated, the centroid of the α-distribution shifted to a value significantly below one, resulting in a simulated distribution that closely resembled the experimentally obtained lipid data (Figure S7B, inset, black). Furthermore, the centroids of the diffusion constant histograms derived from individual trajectories using both AP and IP methods (for regression and non-regression analyses) also shifted toward the experimental centroid values (data not shown), even when the MLR was optimized.

Figure S7C displays a cumulative squared displacement (CSD) analysis of Cy5-DHPE lipid data processed using a calibrated MLR after correction for blur and localization uncertainty. Importantly, only displacements between consecutive frames are utilized (N_p = 1, 42 ms). Cumulative squared displacement assesses how the movement of a particle evolves over time and can identify deviations from normal Brownian motion, or states of Brownian motion, by changes in slope. It captures the accumulated effect of displacement and is less impacted by photobleaching and trajectory noise. Cumulative squared displacement analysis is particularly adept at detecting transient or time-dependent changes in diffusion, such as sudden shifts from confined to free diffusion. Applying CSD to individual trajectories can also reveal both time-dependent and static heterogeneities in individual particle behavior. The individual trajectory analysis in Figure S7C (colored lines) reveals variation in slope; however, these differences fall within the expected ergodic distribution for a single diffusion constant. The results also suggest that some molecules exhibit apparent changes in diffusion behavior over time, as indicated by deviations from linearity at large displacements. This apparent variation arises because the CSD analysis is sensitive to incorrect trajectory linkages at larger step sizes. Nevertheless, the impact of these errors appears minimal and can be eliminated altogether by limiting the analysis to $MLR^2/4t$ (not shown in Figure S7C). When the ensemble average of all trajectories (black) is fit to eq. 12, the resulting diffusion constant agrees closely with values obtained from both FCS and *MSD*-based analyses.

h. Summary of Diffusion Analysis Methodologies and Error Susceptibility

In the preceding section, we compared several *MSD*-based (SS, AP, IP, regression, and non-regression) and CSD-based methods (defined in Sections 2c–f) for quantifying lipid diffusion using tracking algorithms that apply cost-matrix minimization at surface concentrations representative of experimental conditions in microelectrode cavity arrays. The accuracy comparison reveals that methods are variably susceptible to linking errors, which can bias both diffusion constants and the anomalous diffusion exponent. Regression-based single-trajectory analyses using AP or IP protocol are more sensitive to linking errors, as are non-regression AP and IP based methods. Alternatively, the SS *MSD* computation method is more robust. Although also affected by incorrect linkages, the CSD method offers complementary strengths and resists undue influence

from incorrect linkages. When properly corrected for blur and localization uncertainty and paired with an optimal MLR, the SS *MSD*, ensemble regression-based *MSD*, and CSD methods produce the most accurate diffusion estimates.

REFERENCES

(1) Bailey, D. J.; Kindt, J. T.; Taylor, M. M.; Paulson, A. R.; Jones, B. H.; Hubbell, K. L.; Keranen-Burden, L. M.; Burden, D. L. Post-Hoc Vibration Mitigation for Single-Molecule Tracking and Diffusion Measurements in Lipid Membranes. *Spectrosc. Lett.* **2010**, *43* (7–8), 586–596. https://doi.org/10.1080/00387010.2010.510734.

(2) Saxton, M. J. Single-Particle Tracking: The Distribution of Diffusion Coefficients. *Biophys. J.* **1997**, *72*, 1744–1753. https://doi.org/10.1016/S0006-3495(97)78820-9.

(3) Qian, H.; Sheetz, M. P.; Elson, E. L. Single Particle Tracking. Analysis of Diffusion and Flow in Two-Dimensional Systems. *Biophys. J.* **1991**, *60*, 910–921. https://doi.org/10.1016/S0006-3495(91)82125-7.

(4) Martin, D. S.; Forstner, M. B.; Käs, J. A. Apparent Subdiffusion Inherent to Single Particle Tracking. *Biophys. J.* **2002**, *83*, 2109–2117. https://doi.org/10.1016/S0006-3495(02)73971-4.

(5) Goulian, M.; Simon, S. M. Tracking Single Proteins within Cells. *Biophys. J.* **2000**, *79* (4), 2188–2198. https://doi.org/10.1016/S0006-3495(00)76467-8.

(6) Savin, T.; Doyle, P. S. Static and Dynamic Errors in Particle Tracking Microrheology. *Biophys. J.* **2005**, *88*, 623–638. https://doi.org/10.1529/biophysj.104.042457.

(7) Berglund, A. J. Statistics of Camera-Based Single-Particle Tracking. *Phys. Rev. E* **2010**, *82*, 011917. https://doi.org/10.1103/PhysRevE.82.011917.

(8) Kerkhoff, Y.; Block, S. Analysis and Refinement of 2D Single-Particle Tracking Experiments. *Biointerphases* **2020**, *15* (2), 021201. https://doi.org/10.1116/1.5140087.

(9) Wieser, S.; Schütz, G. J. Tracking Single Molecules in the Live Cell Plasma Membrane-Do’s and Don’t’s. *Methods* **2008**, *46*, 131–140. https://doi.org/10.1016/j.ymeth.2008.06.010.

(10) Honciuc, A.; Harant, A. W.; Schwartz, D. K. Single-Molecule Observations of Surfactant Diffusion at the Solution−Solid Interface. *Langmuir* **2008**, *24* (13), 6562–6566. https://doi.org/10.1021/la8007365.

(11) Schütz, G. J.; Schindler, H.; Schmidt, T. Single-Molecule Microscopy on Model Membranes Reveals Anomalous Diffusion. *Biophys. J.* **1997**, *73* (2), 1073–1080. https://doi.org/10.1016/S0006-3495(97)78139-6.
